# On the quest for hidden ovarian teratomas in therapy-refractory anti-NMDA receptor encephalitis: a case report

**DOI:** 10.1186/s42466-022-00181-0

**Published:** 2022-04-25

**Authors:** Christoph Cirkel, Anna Cirkel, Georg Royl, Alex Frydrychowicz, Lars Tharun, Steffen Deichmann, Achim Rody, Thomas F. Münte, Björn Machner

**Affiliations:** 1 Department of Gynecology, University Hospital Schleswig Holstein, Campus Lübeck, Ratzeburger Allee 160, 23538 Lübeck, Germany; 2 Department of Neurology, University Hospital Schleswig Holstein, Campus Lübeck, Ratzeburger Allee 160, 23538 Lübeck, Germany; 3 Department of Radiology, University Hospital Schleswig Holstein, Campus Lübeck, Ratzeburger Allee 160, 23538 Lübeck, Germany; 4 Institute of Pathology, University Hospital Schleswig Holstein, Campus Lübeck, Ratzeburger Allee 160, 23538 Lübeck, Germany; 5 Department of Surgery, University Hospital Schleswig Holstein, Campus Lübeck, Ratzeburger Allee 160, 23538 Lübeck, Germany

**Keywords:** NMDA encephalitis, Ovarian teratoma, Laparoscopy, Oophorectomy

## Abstract

**Background:**

Anti-NMDA-receptor (anti-NMDAR) encephalitis is often associated with ovarian teratoma (OT). The best management of anti-NMDAR encephalitis patients with normal imaging studies (pelvic ultrasound/MRI) but clinically high risk of OT (e.g., female, adult, black) is unclear. We report on the surprising diagnostic quest in a young black woman with anti-NMDAR encephalitis, in whom invasive procedures could finally disclose two OTs that were hidden from the initial non-invasive diagnostics.

**Case report:**

The patient presented with a one-week history of psychotic symptoms, developing oro-facial dyskinesia, seizures and coma, eventually requiring mechanical ventilation. NMDA-receptor antibodies were positive in serum and cerebrospinal fluid. Pelvic MRI and transabdominal ultrasound were normal. Exploratory laparoscopy was also unremarkable at first, but due to a suspicious echogenic mass (15 mm) in the right ovary on perioperative transvaginal ultrasound, an ovarian incision was performed which led to the detection of a first OT and its removal via ovarian-preserving cystectomy. Following a severe therapy-refractory clinical course despite aggressive immunotherapy and tumor removal, 6 months later bilateral oophorectomy was performed as ultima ratio, disclosing a second micro-OT (6 mm) in the left ovary. Unfortunately, the patient has not improved clinically yet.

**Conclusions:**

In therapy-refractory anti-NMDAR encephalitis with high risk of OT, small and bilateral OTs hidden from primary non-invasive diagnostics should be considered, which may trigger further invasive diagnostic procedures.

## Main text

Anti-N-methyl-D-aspartate receptor (anti-NMDAR) encephalitis is frequently (24–38%) associated with an underlying tumor, usually (85–94%) ovarian teratoma (OT) [2, 8]. The likelihood of OT thereby depends on age, sex and ethnicity, being highest (> 50%) in patients who are older than 18 years, female, black or Asian [3, 8]. Outcome is better if an OT is detected and removed early, primarily via ovarian-preserving resection or alternatively via oophorectomy [5, 8]. As there is no serum tumor marker for OT, the recommended screening for OT includes ultrasound and pelvic MRI [9]. The best management of patients with negative imaging studies but high clinical probability of OT is unclear. The options encompass immunotherapy without further search for OT, repetitive screening for OT (e.g., every 6 months), explorative laparoscopy or blind oophorectomy [4, 8, 9].

**Fig. 1:**
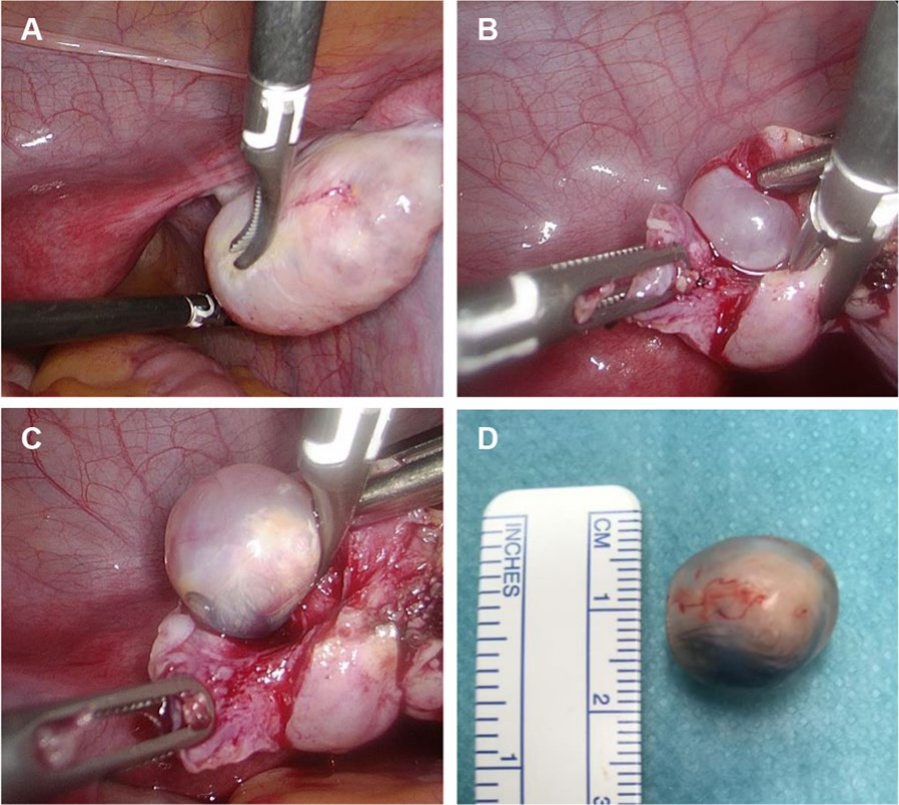
Laparoscopic images of the right ovary. **A** Normal appearance of the right ovary. **B** Incision of the right ovary and detection of a suspicious cyst. **C** Ovarian-preserving preparation of the cystic tumor. **D** Completely resected mature OT (histopathologically confirmed)

We report on a 21 year-old black woman presenting with the characteristic syndrome of anti-NMDAR encephalitis developing psychotic symptoms over one week, followed by oro-facial dyskinesia, seizures, autonomic dysfunction (hypotension, hypersalivation) and loss of consciousness finally requiring intubation and mechanical ventilation. Brain MRI showed subtle T2 signal hyperintensities in the bilateral hippocampus and claustrum. The CSF analysis revealed moderate lymphocytic pleocytosis (71/µl), normal protein, glucose and lactat. Using indirect immunofluorescence, NMDA receptor antibodies were positive in serum (titers 1:1000 cell-based, 1:320 tissue-based assay) and cerebrospinal fluid (titers 1:320 cell-based, 1:100 tissue-based assay). The CRP was slightly increased (9.9 mg/l, reference > 5.0). Apart from that, laboratory studies were normal for basic parameters (complete blood count, hemostasis, electrolytes, liver enzymes, creatinine, TSH) and also negative for further auto-antibodies (CASPR2, GABA b, LGI 1, GAD, AMPA, MOG, Amphiphysin, CV2, PNMA2, Hu, Yo, RI, SOX1, Titin, DPPX and IgLON5). Pelvic MRI and transabdominal ultrasound (TAS) were normal. Considering the patient’s individual high risk for OT, we prepared the patient for explorative laparoscopy and also performed transvaginal ultrasound (TVS) in adequate lithotomy position in the operation room. This revealed a small (15 mm) echogenic mass in the right ovary, while left ovary was unremarkable. During exploratory laparoscopy the right ovary first appeared completely normal (Fig. 1A). However, after incision of the outer capsule a small cystic tumor popped out (Fig. 1B), which could be completely removed with the ovary being preserved (Fig. 1C/D). Histo-pathological investigation confirmed a mature teratoma. The left ovary was equally incised, but no tumor was detected.

**Fig. 2:**
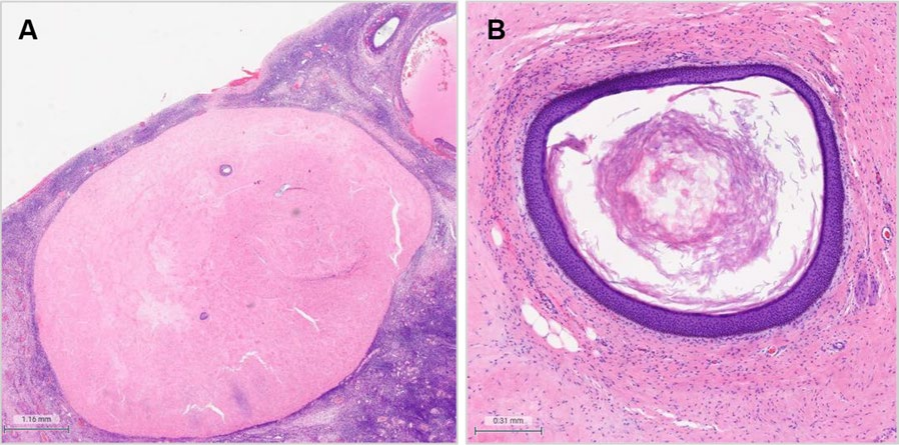
Histopathological images of left ovary. **A** Solid mature teratoma (6 mm) with **B** squamous differentiation. HE stain, 1×/4× magnification

Despite early tumor removal (day 11 of hospitalization), the patient did not improve clinically, the titer of antibodies did not decrease and she developed many complications (status epilepticus, need for permanent sedation and mechanical ventilation, septic shock). Immunotherapy included corticosteroids (methylprednisolone iv 500 mg/d over the first 5 days, followed by oral prednisolone 1 mg/kg per day, slowly tapered to 7,5 mg/days maintenance dose), plasma exchange (3 cycles of a daily plasmapheresis over five consecutive days; applied in week 1, 5 and 9 after the initial admission), Rituximab (2 cycles: 1000 mg in week 3 and in week 7) and Bortezomib (2 cycles, each with 1.3 mg/m^2^ administered on days 1, 4, 8, and 11; conducted in month 4 and month 6 with a prolonged pause in between due to septic shock) [7]. Due to the severe clinical course refractory to aggressive immunotherapy and tumor removal, the patient finally underwent bilateral oophorectomy (6 months after admission). Although both ovaries appeared macroscopically normal, histopathological examination disclosed a second, very small (6 mm) teratoma in the left ovary (Fig. 2). Unfortunately, despite bilateral oophorectomy (month 6) and application of four more cycles of Bortezomib (between month 7 and 10) and four cycles of Cyclophosphamid (1145 mg each, applied between month 10 and 12), the patient is still requiring deep sedation and mechanical ventilation for seizure control, twelve months after disease onset.

This patient with severe anti-NMDAR encephalitis revealed clinical characteristics (female, adult, black) pointing to an especially high risk of OT [3, 8]. Despite normal pelvic MRI and TAS we therefore performed exploratory laparoscopy including ovarian incision and perioperative TVS. The abnormal finding in the right ovary might indicate higher OT sensitivity of TVS than TAS/MRI, but comparative studies are missing [9]. Furthermore, intensive care units are often not sufficiently equipped for adequate positioning of patients during TVS and the ultrasound devices may lack a transvaginal probe. Therefore, in patients requiring intensive care, we advise performing TVS under optimal conditions, e.g., in the operation room. Concerning laparoscopy, an incision of the right ovary was necessary to detect the (first) OT, which would not have been disclosed by ‘non-invasive’ exploration. Considering the patient’s young age and future fertility [5], we initially performed ovarian-preserving cystectomy, which represents the standard-of-care in treating OTs. However, the further course with lack of clinical improvement, severe complications and permanently increased antibodies despite tumor removal and aggressive immunotherapy finally made the oophorectomy inevitable. This ‘blind’ oophorectomy revealed the second OT. Although usually unilateral, bilateral OTs occur in about 11% of OT-associated anti-NMDAR encephalitis cases [1]. It remains a difficult and individual clinical decision whether or not bilateral oophorectomy should be conducted in therapy-refractory cases, because a tumor is not always detected and clinical improvement, as in our case, cannot be guaranteed [3, 4, 6]. Prior to oophorectomy, fertility preservation via ovarian stimulation and oocyte cryopreservation might be considered but our patient’s critical state did not allow for such procedures.

In conclusion, if non-invasive diagnostics (pelvic MRI/TAS) are negative in anti-NMDAR encephalitis patients with OT high risk, we first recommend TVS and even if negative an exploratory laparoscopy with close inspection of the ovaries. Our case further suggests that in patients with a severe therapy-refractory course, bilateral oophorectomy should be taken into consideration, even when a first OT has been removed via ovarian-preserving cystectomy, because a second (micro-)OT sustaining anti-NMDAR encephalitis remains possible.

## Data Availability

Anonymized data not published within this article will be made available upon reasonable request.
